# Detection of Small Magnetic Fields Using Serial Magnetic Tunnel Junctions with Various Geometrical Characteristics

**DOI:** 10.3390/s20195704

**Published:** 2020-10-07

**Authors:** Zhenhu Jin, Yupeng Wang, Kosuke Fujiwara, Mikihiko Oogane, Yasuo Ando

**Affiliations:** 1 Department of Applied Physics, Tohoku University, Sendai 980–8579, Japan; wang.yupeng.r6@dc.tohoku.ac.jp (Y.W.); oogane@mlab.apph.tohoku.ac.jp (M.O.); ando@mlab.apph.tohoku.ac.jp (Y.A.); 2 Spin Sensing Factory Corporation, Sendai 980–0845, Japan; kosuke.fujiwara@spintronics.co.jp; 3 Center for Science and Innovation in Spintronics (Core Research Cluster), Tohoku University, Sendai 980–8577, Japan; 4 Center for Spintronics Research Network, Tohoku University, Sendai 980–8579, Japan

**Keywords:** tunnel magnetoresistance, magnetic tunnel junction, magnetoresistance sensor, integrated magnetic sensor

## Abstract

Thanks to their high magnetoresistance and integration capability, magnetic tunnel junction-based magnetoresistive sensors are widely utilized to detect weak, low-frequency magnetic fields in a variety of applications. The low detectivity of MTJs is necessary to obtain a high signal-to-noise ratio when detecting small variations in magnetic fields. We fabricated serial MTJ-based sensors with various junction area and free-layer electrode aspect ratios. Our investigation showed that their sensitivity and noise power are affected by the MTJ geometry due to the variation in the magnetic shape anisotropy. Their MR curves demonstrated a decrease in sensitivity with an increase in the aspect ratio of the free-layer electrode, and their noise properties showed that MTJs with larger junction areas exhibit lower noise spectral density in the low-frequency region. All of the sensors were able detect a small AC magnetic field (*H*_rms_ = 0.3 Oe at 23 Hz). Among the MTJ sensors we examined, the sensor with a square-free layer and large junction area exhibited a high signal-to-noise ratio (4792 ± 646). These results suggest that MTJ geometrical characteristics play a critical role in enhancing the detectivity of MTJ-based sensors.

## 1. Introduction

The measurement of small, low-frequency magnetic fields is critically important in several industrial fields [1,2,3]. Magnetoresistive (MR) sensors, such as anisotropic magnetoresistance (AMR), giant magnetoresistance (GMR), and tunnel magnetoresistance (TMR) sensors, are considered promising candidates for magnetic sensor applications. Compared with conventional sensors, such as the induction coil and Hall-effect sensor, MR sensors have the advantages of high sensitivity, low power consumption, and small size [4,5]. In particular, thanks to their extremely high magnetoresistance at room temperature, MgO-barrier magnetic tunnel junction (MTJ)-based TMR sensors have attracted interest for detecting small magnetic fields in various fields including medical diagnosis, biosensing, and electromagnetic nondestructive testing [6,7,8,9,10,11,12,13]. In a MTJ element, two ferromagnetic layers are separated by a thin insulator layer (nanometer order) and follow a spin current perpendicular to the plane configuration. One ferromagnetic layer (free layer (FL)) is magnetically soft and the other (pinned layer) is magnetically hard. If a bias voltage is applied between the two ferromagnetic layers, the spin currents can tunnel through the insulator layer due to a spin tunneling effect, and the electrical resistance is lower when both magnetic moments are aligned in parallel and higher when they are aligned in an anti-parallel configuration. Therefore, the MTJ output can reflect the external field strength. For magnetic sensor applications, improvement of the sensitivity and reduction of the noise are required to ensure a high signal-to-noise ratio (SNR). The sensitivity is determined by TMR/2*H_k_* (or Δ*V*/2*H_k_*), where *H_k_* is a magnetic anisotropy of the FL. Many studies have reported that MTJs with half-metals, such as Heusler alloys, can exhibit a high TMR ratio due to their high spin polarization [14,15,16]. However, optimization of the interface mismatch between Heusler alloys and the MgO barrier is still necessary to further enhance the TMR ratio. In addition, the sensitivity could be increased by suppressing the saturation field when the TMR sensors are incorporated with the magnetic flux concentrator [17,18], though further enhancement of sensitivity is difficult due to the limited sensing area for sensor application. Since the *H_k_* is correlated with the geometry of the FL, TMR curves, as well as their sensitivity, can be tuned by changing the shape and size of MTJs [19,20,21]. Optimizing the MTJ geometrical characteristics, therefore, shows promise as a way to improve sensor performance. Reducing the 1/*f* noise in measurements is also important to gain a high signal-to-noise ratio. The noise power in MTJs is mainly determined by several mechanisms, including shot noise at a nonzero bias voltage, thermal noise at room temperature, and 1/*f* noise (electrical 1/*f* noise and magnetic 1/*f* noise) [22,23]. Previous studies had been reported that integrated MTJ-based sensors provide excellent detectivity due to the reduced electrical 1/*f* noise in MTJs with an array configuration [24,25,26]. However, resistance fluctuations and magnetic noise are inevitably produced during the reversal of magnetization in the ferromagnetic layer of MTJs. Considering the significant effect of magnetic anisotropy in the magnetization reversal process in MTJs, an MTJ with various geometrical characteristics could show different noise properties and, therefore, different SNRs. In the present study, to improve the detectivity of MTJ-based sensors, we fabricated sensors based on 20 serial MTJs with various junction areas and aspect ratios and then experimentally investigated their resistance response and noise characteristics. We also investigated their output signals to determine the maximum signal-to-noise ratio that can detect a low-frequency magnetic field.

## 2. Experimental Methods

**Figure 1:**
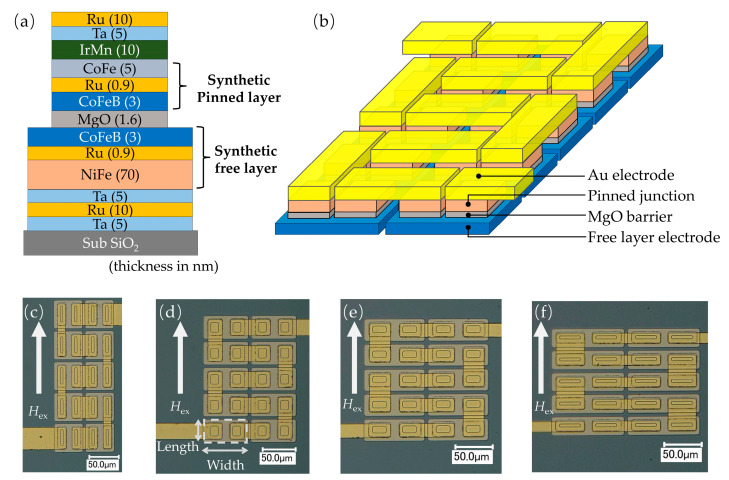
(a) Magnetic film structure. (b) Schematic diagram of 20 serial MgO-barrier magnetic tunnel junctions (MTJs). (c)–(f) Microscopic images of 20 serial MTJs with different junction areas (length × width = 15 μm × 40 μm; 23 μm × 26 μm; 30 μm × 20 μm; 40 μm × 15 μm) and footprint areas of the free layer (length × width = 50 μm × 50 μm; 70 μm × 36 μm; 86 μm × 29 μm; 100 μm × 25 μm) with various free layer pattern (FL electrode) aspect ratios. White arrows denote the direction of external magnetic fields.

MTJ films were prepared using an ultrahigh vacuum magnetron sputtering system (pressure of less than 3×10^−6^ Pa). As shown in Figure 1a, the film structure was Si/SiO_2_/Ta(5)/Ru (10)/Ta (5)/Ni_80_Fe_20_ (70)/Ru (0.9)/Co_40_Fe_40_B_20_ (3)/MgO (1.6)/Co_40_Fe_40_B_20_ (3)/Ru (0.9)/Co_75_Fe_25_ (5)/Ir_22_Mn_78_ (10)/Ta (5)/Ru (20) (in nm). The Ni80Fe20 (70 nm) and Co40Fe40B20 (3 nm) layers were antiferromagnetically coupled via the Ru (0.9 nm) layer. Owing to the thick NiFe layer, the magnetization switching of the bottom CoFeB layer occurred simultaneously with that of the NiFe layer. The serial MTJs were microfabricated using photolithography and argon ion milling. By using an ion milling probe (IMP) end point detector, the milling process could be observed and stopped in the middle of the barrier. Figure 1b shows the serial MTJ configuration, where two pinned junctions were fabricated on a continuous FL electrode. The top Au electrodes were deposited to connect the pinned junctions in series, after which the applied current could flow into each junction. We fabricated three series of serial MTJs with different areas and shapes, as listed in Table 1. For each series, the aspect ratio (length/width) of the ferromagnetic layer pattern (FL electrode) was varied from 1 to 4, and a total of 12 types of serial MTJs were fabricated.

After microfabrication, a two-step annealing process was used to obtain a high magnetoresistance and linear resistance response. The first annealing process was carried out at 350 ℃ for 1 h in a magnetic field of 10 kOe to induce the magnetic anisotropy of the FL and obtain a high TMR ratio due to coherent Δ1 tunneling at the crystallized Co40Fe40B20/MgO interface [27,28]. The second annealing process was carried out at 300 ℃ for 1 h with a 90° rotated magnetic field of 10 kOe for rotation in the easy axis direction of the pinned layer. After the second annealing, the MTJ was able to offer a linear magnetoresistance response due to the orthogonal easy axis of the free and pinned layers [29]. 

**Figure 2:**
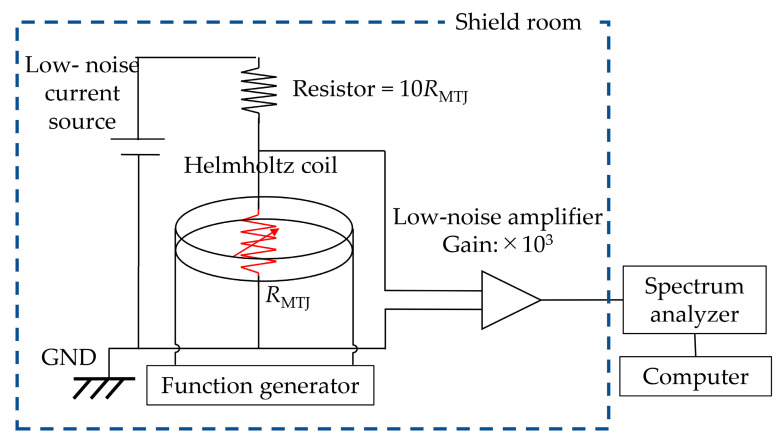
Schematic diagram showing detection of the AC magnetic field using an MTJ sensor.

To determine the magnetoresistance characteristics of the MTJ sensors, we obtained magnetoresistance transfer curves using the DC four-probe method with a uniform magnetic field of ±90 Oe at room temperature. The direction of the applied field was the same as that of the pinned direction. To determine detectivities, the MTJ sensors were used to detect a weak magnetic field in a magnetically shielded room that protected them from geomagnetism and urban noise, as well as to provide a low magnetic field environment (<1 mOe). Figure 2 shows a schematic view of the measurement setup. The Helmholtz coil was connected to a function generator, and a 23 Hz sine wave was applied to the Helmholtz coil to induce a uniform magnetic field (*H*_rms_ ≈ 0.3 Oe). A low noise amplifier (SR560) with a gain of 1000 was used to maximize the outputs. A spectrum analyzer (E4448A) was used to acquire the digitized output signal. 

## 3. Sensitivity and Noise Power

**Figure 3:**
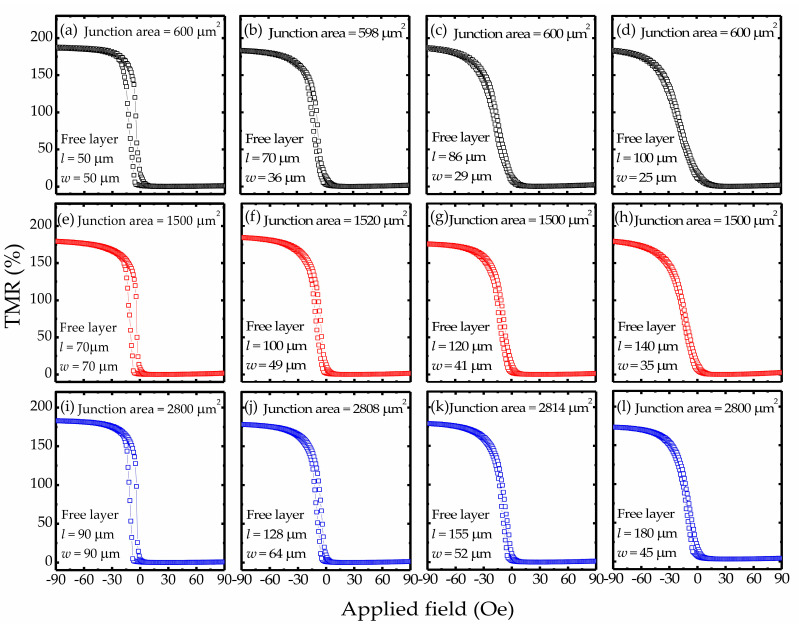
Magnetoresistance transfer curves for MTJ sensor Series A (**a**–**d**), B (**e**–**h**), and C (**i**–**l**) at a bias voltage of 100 mV and zero magnetic field. The aspect ratios of the free layer electrodes ranged from 1 to 4.

**Figure 4:**
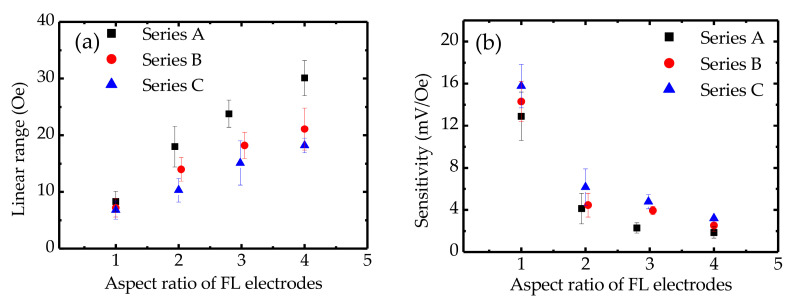
Dependence of (**a**) the linear range of the resistance response and (**b**) sensitivity on the aspect ratio of the free layer that ranged from 1 to 4 at a bias voltage of 100 mV and zero magnetic field. Here, the linear range shows a nonlinearity of 10% FS for each sensor.

Figure 3a–l shows the magnetoresistance transfer curves (resistance versus applied field) for fabricated MTJ sensors. The measurements were performed with a bias voltage of 100 mV. All sensors showed high TMR ratios (above 172%) and linear resistance responses. The junction areas ranged from 598 to 2808 μm^2^, and the MTJ sensors showed *R*_p_*A* (MTJ resistance in the parallel-magnetization state × junction area) values of about 9.6 × 10^6^ Ωμm^2^ for all series. These measurement results show that the MTJ sensors with various geometrical characteristics exhibit different TMR curves, which indicates that varying the shape and size of an MTJ can result in variation of the resistance response. In general, magnetization switching is strongly dependent on the geometrical characteristics of the MTJ due to magnetic shape anisotropy [19,30,31]. Changing the shape of the ferromagnetic layer electrode can effectively tune the linear range in TMR curves as well as the sensitivity. When a magnetic field is applied in the direction of the short side of an FL electrode (pinning direction), the demagnetization field is induced, and its factor is associated with the in-plane shape of the ferromagnetic layers. Therefore, MTJs with a high aspect ratio exhibit a high anisotropy field. This phenomenon can be observed from the dependence between the linear range and the FL aspect ratio. Figure 4a shows the linear range of the resistance response plotted as a function of the FL aspect ratio and clearly indicates that a higher FL electrode aspect ratio gives a wider linear range for the resistance response and smaller hysteresis. Although Series A, B, and C sensors have different junction areas, their TMR curves indicate a tendency for the linear range to increase as the FL aspect ratio increases. Correspondingly, as shown in Figure 4b, the sensor sensitivity, which is determined by the slope of the TMR curves under a zero field, decreases when the FL electrode aspect ratio increases in a range from 1 to 4 due to the increased demagnetization in the ferromagnets. For this reason, MTJ sensors with a low FL electrode aspect ratio show higher sensitivities than the other ones. The sensor with FL electrodes of 100 μm × 100 μm showed a small linearity range for the resistance response. However, it exhibited a considerably high sensitivity value, over 15.7 ± 2.1 mV/Oe, at a bias voltage of 100 mV. 

**Figure 5:**
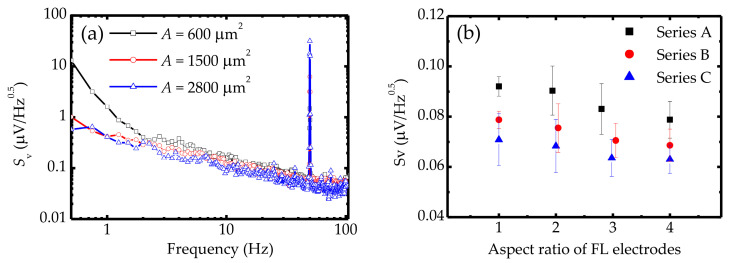
(**a**) Noise spectral density *S*_v_ as a function of frequency for serial MTJs with various pinned junction areas at a bias voltage of 100 mV under a magnetic field of 0 Oe. (**b**) Relationships among various geometrical characteristics of serial MTJs and noise spectral density at a certain frequency (23 Hz).

In MTJs, the noise power is an important factor for the measurement of external fields. The total noise spectrum can be expressed by [23]

(1) $$S_{B}^{Total}={\left(dB/dV\right)}^{2}\left[S_{V}^{Amp}+S_{V}^{therm-shot}+S_{V}^{elec,\frac{1}{f}}+S_{V}^{RTN}\right]+S_{B}^{therm,mag.}+S_{B}^{mag.1/f}$$

where $S_{V}^{Amp}$ is the amplifier noise, $S_{V}^{therm-shot}$ is the thermal-shot noise, $S_{V}^{RTN}$ is the random telegraph noise, $S_{B}^{therm,mag.}$ is the thermal magnetic noise, and $S_{V}^{elec,1/f}$ and $S_{B}^{mag.1/f}$ are the electronic and magnetic 1/*f* noise, respectively. Here, white noise contains $S_{V}^{Amp}$, $S_{V}^{therm-shot}$, and $S_{B}^{therm,mag.}$, which is independent of frequency. The $S_{V}^{RTN}$ is induced from the charging and discharging process of the defect center, and it can be eliminated by using a proper annealing process [32]. Hence, frequency-dependent noise is a dominant noise source in the low-frequency region and its mechanism can be divided into two parts: Electrical 1/*f* noise $S_{V}^{elec,1/f}$ and magnetic 1/*f* noise $S_{B}^{mag,1/f}$. The $S_{V}^{elec,1/f}$ is ubiquitous low-frequency noise in metallic films, and its origin can be attributed to the charge trapping of electrons in barriers and the ferromagnetic layer/barrier interface. The $S_{V}^{elec,1/f}$ in an MTJ can be expressed as

(2) $$S_{V}^{elec,1/f}=\alpha_{elec}V^{2}/\left(Af^{\beta }\right)$$

where *α*_elec_ is a Hooge parameter of electrical 1/*f* noise, *V* is the bias voltage, and *A* is the junction area. With a bias voltage, the $S_{V}^{elec,1/f}$ can be reduced by increasing *A*. Therefore, as shown in Figure 5a, MTJs with a large junction area (*A* ≈ 2800 μm^2^) exhibited a relatively low noise spectral density in the low-frequency region. In addition to electrical 1/f noise, the magnetization state also affects the noise behavior at the low-frequency region. Previous studies have shown that magnetic-relative noise does not only depend on the frequency but also on the external magnetic field [33,34,35]. Consequently, magnetic noise is associated with the magnetoresistance response, and a larger d*R*/d*H* term slope under a zero field inevitably gives a higher low-frequency noise power. As shown in Figure 5b, by applying the same voltage to each series of MTJs, the noise spectral density at the low-frequency region slightly decreases as the FL aspect ratio increases. This result explains how the dependence of the magnetic noise power decreases as the field sensitivity increases. Therefore, a higher noise spectral density can be observed for MTJs with a square FL electrode compared with the ones with other sensors. Additionally, an increase in the junction area can suppress $S_{V}^{elec,1/f}$, resulting in large-area MTJ-based sensors (Series C) that exhibit relatively low noise power, as shown in Figure 5b. These results demonstrate that Series C sensors can provide excellent detectivity owing to their high sensitivity and low noise power. 

## 4. Detection of a Certain Low-Frequency Magnetic Field

**Figure 6:**
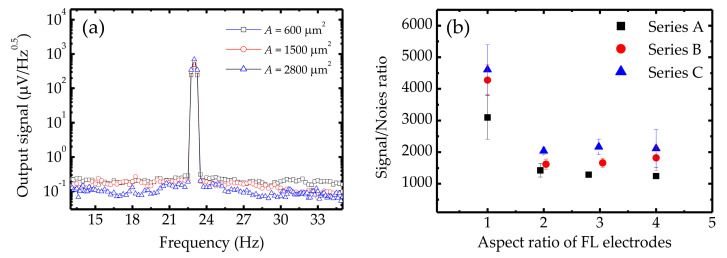
(**a**) Output signals from serial MTJs with same aspect ratio of free layer but different junction area *A*, at a bias voltage of 100 mV. (**b**) SNR (*S*_peak_/*S*_background_) from the detection of magnetic field of 0.3 Oe using MTJs with various dimensional characteristics. Aspect ratios of the free layer electrode ranged from 1 to 4.

Figure 6a shows an AC magnetic field that was detected by an MTJ with the same free-layer shape but different junction areas. Clear voltage peaks can be observed at the MTJ output where a 23-Hz magnetic field was applied from a Helmholtz coil. All sensors detected the low-frequency AC magnetic field and provided high SNR (*S*_peak_/*S*_background_). Particularly, serial MTJs with square FL electrodes can exhibit high SNRs (4610 ± 781), which indicates that their estimated RMS value of detectable AC-magnetic field is approximately 65 μOe at 23 Hz with an SNR of 1. Figure 6b shows the SNRs of Series A, B, and C sensors when sensing the external AC magnetic field. We can see that the geometrical characteristics of the MTJs had a significant effect on the detectivity of the sensors. For MTJs with the same FL aspect ratio, those with larger junction areas exhibited higher SNRs, which indicates that increasing the junction area is a feasible solution for achieving excellent MTJ detectivity. Furthermore, the SNR rapidly decreased when the FL aspect ratio was increased from 1 to 2, which contributed to the dramatically decreased sensitivity. However, due to a slightly higher hysteresis, the sensor with a square FL electrode showed a larger error in the SNR value. After the aspect ratio above 2, the SNR value became saturated and showed a smaller error bar owing to the lower hysteresis. Since the sensitivity and noise slightly decreased when the FL aspect ratio increased, when the aspect ratio ranged from 2 to 4, the sensors in individual series exhibited similar SNR values. 

## 5. Conclusion

In this study, we fabricated and characterized serial MTJs with various FL shapes and junction areas. TMR curves showed that the linear ranges and sensitivities were closely dependent on the MTJ geometry. The sensors with a square FL and large junction area exhibited extremely high sensitivity due to their low magnetic shape anisotropy. Moreover, increasing junction area *A* significantly reduced the noise power in the low-frequency region. All of the sensors were able to detect a small low-frequency magnetic field (*H*_rms_ = 0.3 Oe). The sensor containing MTJs with a large *A* (*A* ≈ 2800 μm^2^) and a low FL electrode aspect ratio provided the highest signal-to-noise ratios, contributing to their high sensitivity. Overall, the experimental results demonstrate that the magnetic shape anisotropy in serial MTJs plays a significant role in detecting magnetic field variations. The MTJs with a square FL electrode had small linearity ranges and little high hysteresis in the resistance response, making them unsuitable for sensor applications. In contrast, MTJs with a low-aspect-ratio FL electrode exhibited high sensitivity when detecting imperceptible fields. Additionally, we found that for sensing small magnetic fields that do not require an extremely high spatial resolution, increasing the junction area is a feasible approach for achieving excellent detectivity for MTJ-based sensors.

## Figures and Tables

**Table 1:** Dimensional characteristics of serial MTJ sensors.

Sensor	Free Layer Pattern (FL Electrode) Area (Unit: μm) (Junction Area)			
**Series A sensors**	50×50 (15×40)	70×36 (23×26)	86×29 (30×20)	100×25 (40×15)
**Series B sensors**	70×70 (25×60)	100×49 (38×40)	120×41 (50×30)	140×35 (25×60)
**Series C sensors**	90×90 (35×80)	128×64 (54×52)	155×52 (67×42)	180×45 (80×35)
